# Prognostic significance of preoperative C-reactive protein to albumin ratio in non-small cell lung cancer patients: A meta-analysis

**DOI:** 10.3389/fsurg.2022.1056795

**Published:** 2023-01-06

**Authors:** Dingxiu He, Yong Yang, Yi Yang, Xiaoqu Tang, Kaisen Huang

**Affiliations:** ^1^ Department of Emergency, Deyang People's Hospital, Sichuan, China; ^2^ Department of Cardiology, Deyang People's Hospital, Sichuan, China

**Keywords:** c-reactive protein to albumin ratio, prognosis, meta-analysis, non-small cell lung cancer, surgery

## Abstract

**Objective:**

We aimed to assess whether C-reactive protein to albumin ratio (CAR) is associated with the clinicopathology and prognosis of patients with non-small cell lung cancer (NSCLC) after surgery.

**Methods:**

Several literature databases were searched for eligible studies in English and Chinese published before September 1, 2022, according to the inclusion and exclusion criteria. The pooled odds ratios (ORs) with 95% confidence interval (CI) were calculated to assess the association of CAR in lung cancer with clinicopathological characteristics including age, sex, smoking status, lymph node metastasis, and American Association of Cancer (AJCC) stage. The pooled hazard ratios (HRs) with 95% CI were calculated to assess the association of CAR with prognosis in lung cancer. Publication bias was assessed using Egger's test.

**Results:**

Overall, 9 studies involving 3,359 NSCLC patients were included in this meta-analysis. The CAR was observed to be higher in males, smokers, and patients with lymph node metastasis and correlated with advanced AJCC stage but not with age. Moreover, a high CAR correlated with poor survival. No publication bias was observed in this meta-analysis.

**Conclusions:**

CAR was observed to be a significant biomarker for prognosis and associated with clinicopathological characteristics in patients with NSCLC after surgery.

## Introduction

Lung cancer has the highest incidence rate among all types of cancer. In the United States, it causes more than 350 deaths per day, which is more than the cumulative deaths caused by breast, prostate, and pancreatic cancers. The number of deaths per day caused by lung cancer is 2.5 times more than that caused by colorectal cancer (the second leading cause of cancer-related death in the United States). Lung cancer ranks second in terms of new cases of cancer detected worldwide (1). In China, lung cancer is the most common type of cancer and leading cause of cancer-related death. The age-standardized mortality rate of lung cancer in China is higher than the global average (2). Despite advances in the diagnosis, treatment, and management of lung cancer, the prognosis of patients with lung cancer is still quite unsatisfactory. A retrospective study from China reported that the median survival time of patients with lung cancer is only approximately 1 year (3). Therefore, prior to treatment, the evaluation of effective and reliable prognostic factors is helpful to assess the prognosis of patients with lung cancer and formulate appropriate treatment strategies.

Many studies have reported that age, sex, treatment, and particularly, tumor node metastasis (TNM) stage are independent prognostic factors of lung cancer (4–6). However, patients with lung cancer at the same TNM stage may have different prognoses. Therefore, it is necessary to identify more reliable prognostic markers to guide individualized precise treatment in lung cancer. Some studies have reported that systemic inflammatory response and nutritional status of patients have a considerable impact on the occurrence, development, and metastasis in lung cancer (7–9).

C-reactive protein (CRP) and albumin can represent the inflammatory response and nutritional status in a better way, and studies have confirmed that high CRP and low albumin levels are significantly correlated with the poor prognosis of patients with lung cancer (10, 11). CRP to albumin ratio (CAR) has been confirmed as an independent prognostic factor in patients with gastric, esophageal, or pancreatic cancers (12–14). Some previous studies have explored the relationship between CAR and the clinical characteristics and prognosis in lung cancer. To further explore the consistency of evidence and differences among relevant studies, in this meta-analysis, we aimed to review relevant literature to verify the association between CAR and the clinicopathological characteristics and prognosis of patients with lung cancer.

## Patients and methods

### Search strategy

Literature databases such as PubMed, Embase, Web of Science, Cochrane Library, China National Knowledge Infrastructure (CNKI), China Science and Technology Journal, and Wanfang Database were used to search for the studies in English and Chinese published before September 1, 2022. Keywords retrieved were non-small cell lung cancer (NSCLC) and its medical subject headings, as well as C-reactive protein/albumin ratio, C-reactive protein to albumin ratio, C-reactive protein albumin ratio, CRP/Alb ratio, CAR. Boolean logic operators were used for retrieval according to the requirements of various databases. The languages of studies were limited to English and Chinese PRISMA checklist are shown in Supplementary Materials.

### Inclusion and exclusion criteria

The inclusion criteria were as follows: 1. International peer-reviewed and published observational studies on the relationship between CAR value before surgery and prognosis in NSCLC; 2. Patients with lung cancer were diagnosed by definite pathological diagnosis and not by imaging, laboratory examination, or clinical characteristics; 3. Studies in which a clear cutoff value was determined according to the CAR value so that the patients with NSCLC were divided into high or low CAR groups; and 4. Studies containing sufficient clinical information to extract and calculate the odds ratio (OR) and 95% confidence interval (CI) in patients in high and low CAR groups and those providing sufficient follow-up information to obtain the corresponding hazard ratio (HR) and 95% CI. The HR and CI of overall survival (OS) and recurrence-free survival (RFS) must be corrected for confounding factors using multivariate Cox regression analysis. The exclusion criteria were as follows: 1. Studies containing subjects with nonprimary lung cancer, such as a metastatic tumor, recurrent cancer, and multiple primary cancers; 2. Reviews, case reports, conference abstracts, comments, and other nonoriginal research articles; and 3. Studies in which research object was not human. In case of studies with the same research results, we selected the most complete or latest clinical study.

### Data extraction

The aforementioned databases were fully searched to obtain the preliminary research literature, and duplicate studies were excluded. After the initial screening, we read the title and abstract to further eliminate studies that did not conform to the inclusion criteria of this meta-analysis. The remaining literature was further screened according to the inclusion and exclusion criteria by reading the complete text. For this, two researchers independently extracted and further cross-checked data to make the final decision. After studying all the included literature, basic information of the included literature was collected. Relevant data of patients in high and low CAR groups was extracted at the same time. Differences regarding the data were discussed and resolved by the two researchers, and a third researcher assisted when necessary.

### Quality evaluation

Since all the retrieved literature included retrospective case-control studies, their quality was evaluated according to The Newcastle-Ottawa Scale (NOS) (15). The indicators of this evaluation included the following three aspects: whether the research objects included in the literature were reasonable, whether the comparability among the study groups in the literature was sufficient, and whether the exposure assessment of the research objects included in the literature was the same. The included literature was evaluated and scored using the NOS scale based on the aforementioned three aspects; the higher the score, the better the quality of the literature.

### Statistical analyses

All data were processed and analyzed using Stata 15.0 (64 bit) software. The correlation between CAR and prognosis and clinicopathological features of patients with lung cancer was evaluated using pooled HR and 95% CI. The *Z*-test was used to determine the statistical difference. *P* < 0.05 was considered significant. The heterogeneity of studies was assessed using I^2^ and *Q* tests (16). The disadvantage of the *Q*-test is that it is sensitive to the number of studies. When the sample size of a study is small, the test efficiency is low; false negatives occur easily, and the distribution of heterogeneity cannot be tested. The *I*^2^ test can represent the proportion of heterogeneity in the total variation even when the number of studies is small. Therefore, in this meta-analysis, *Q*-test combined with the I^2^ test was used as the evaluation of heterogeneity. When the heterogeneity was statistically significant, the random effect model was used (*P* < 0.05, *I*^2^ > 50%), otherwise, the fixed-effect model was adopted (*P* ≥ 0.05, *I*^2^ ≤ 50%). When significant heterogeneity was observed, the source of heterogeneity was explored through sensitivity analysis, and the stability of the results was evaluated. Sensitivity analysis refers to comparing the combined effects after excluding the included literature one by one to explore whether the source of heterogeneity is caused by a certain study and to verify the stability of the results. The publication bias was assessed using Egger's test (17). If the Egger plot is symmetrical or Egger's test is not statistically significant (*P* > 0.05), it indicates that there is no publication bias.

## Results

### Literature search and quality evaluation

**Figure 1:**
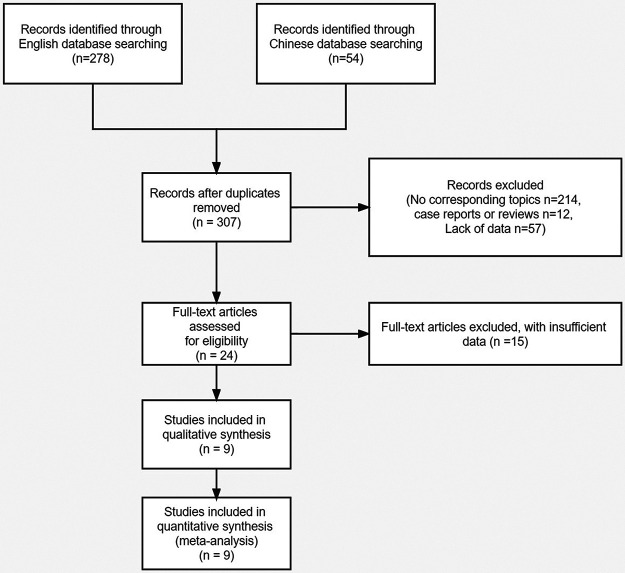
Flow diagram of the literature selection process.

According to the developed search formula, 307 studies were obtained after excluding the studies with repeated results (Figure 1). The titles and abstracts of the included studies were assessed, and 283 studies irrelevant for this meta-analysis were excluded. The remaining studies were completely read, and 15 studies that did not provide available data were excluded. Finally, 9 studies (18–26) (7 in English and 2 in Chinese) that met the inclusion and exclusion criteria were included in this meta-analysis. Table 1 summarizes the basic characteristics of the included studies. The 9 studies in this meta-analysis included 3,359 patients with NSCLC. The maximum follow-up time of each study ranged from 12 to 132 months. The cut-off value of CAR ranges from 0.014 to 0.424. The HR values of all included studies were obtained using multivariate Cox regression analysis. In addition, all included studies with NOS scores ≥ 7 were considered as high-quality studies (Table 2).

**Table 1:** Basic clinical characteristics of the included studies.

First author	Year	Language	Country	The number of patients	Age	Sex (Male/Female)	Cancer type	TNM stage	Cut-off value of CAR	Period	OS			RFS		
											HR	95%CI low	95%CI high	HR	95%CI low	95%CI high
Miyazaki T	2017	English	Japan	109	82 (80–93)	69/39	NSCLC	I-IV	0.028	2003-2014	2.13	1.07	4.30	–	–	–
Yamauchi Y	2017	English	Germany	156	62 ± 10	77/79	Lung adenocarcinoma	IIIA-N2	0.6	2010–2014	–	–	–	4.2	1.14	15.51
Zhang F	2017	English	China	617	60 (30–82)	461/156	NSCLC	I-III	0.424	2006–2009	1.87	1.41	2.49	1.54	1.1	2.16
Zhang X	2019	Chinese	China	100	NA	60/40	NSCLC	I-III	0.25	2014–2018	1.367	0.858	2.177	–	–	–
Kobayashi S	2020	English	Japan	492	69 (31–90)	313/179	NSCLC	I-III	0.04	2009–2014	1.81	1.01	3.25	–	–	–
Fu Q	2021	Chinese	China	284	67 (27–84)	195/89	Lung squamous cell carcinoma and Lung adenocarcinoma	I-III	0.092	2015–2016	1.908	1.075	3.387	–	–	–
Matsubara T	2021	English	Japan	596	69 (34–87)	316/280	NSCLC	I-III	0.016	2010–2015	2.06	1.07	3.98	1.84	1.07	3.17
Watanabe K	2021	English	Japan	387	70.6 ± 9.4	233/154	NSCLC	I-III	0.014	2010–2019	–	–	–	1.987	1.202	3.284
Miyazaki T	2022	English	Japan	618	82 (81–84)	353/265	NSCLC	I	0.106	2–15–2016	2.16	1.22	3.83	–	–	–

**Table 2:** Study quality and bias in the retrospective cohort studies judged by the Newcastle-Ottawa scale (NOS) checklist.

First author	Total score	Cohort selection				Comparability	Outcome		
		Representativeness of the Exposed Cohort	Selection of the Non-Exposed Cohort	Ascertainment of Exposure	Demonstration that outcome of interest was not present at start of study	Comparability of cohorts on the basis of the design or analysis	Assessment of outcome	Was follow-up long enough for outcomes to occur	Adequacy of follow up of cohorts
Miyazaki T	8	★	★	★	★	★	★	★	★
Yamauchi Y	9	★	★	★	★	★★	★	★	★
Zhang F	8	★	★	★	★	★	★	★	★
Zhang X	7	★	★		★	★	★	★	★
Kobayashi S	8	★	★	★	★	★	★	★	★
Fu Q	9	★	★	★	★	★★	★	★	★
Matsubara T	8	★	★	★	★	★	★	★	★
Watanabe K	8	★	★	★	★	★	★	★	★

*p < 0.05.

### Association between CAR and clinicopathological features in patients with NSCLC

**Figure 2:**
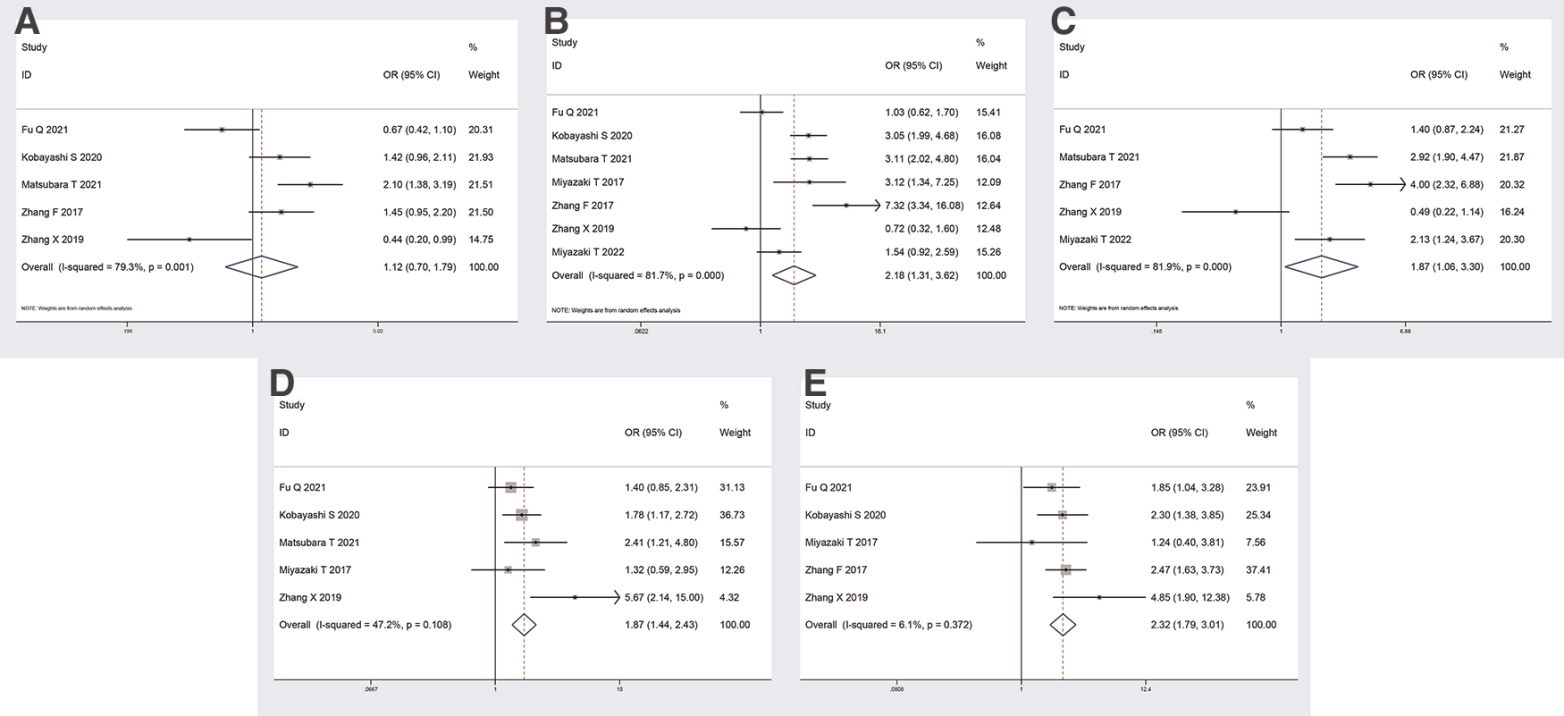
Forest plot of the CAR and clinicopathological characteristics. Forest plot evaluating the ORs of the correlation between CAR and (**A**) age, (**B**) sex, (**C**) smoking status, (**D**) lymph node metastasis, and (**E**) AJCC stage. OR, odds ratio; CAR, C-reactive protein to albumin ratio; AJCC, American Association of Cancer.

The analyses of the association between CAR and clinicopathological characteristics are shown in Table 3 and Figures 2A–E. A combination of 5 studies using a random-effects model suggested no significant association between age and CAR (OR = 1.122, 95% CI = 0.703–1.792, *P* = 0.629). A significant association existed between sex and CAR; men exhibited higher CAR as observed after combining 7 studies using the random-effects model (OR = 2.181, 95% CI = 1.313–3.620, *P* = 0.003). Patients who smoke exhibited higher CAR after combining 5 studies using the random-effects model (OR = 1.870, 95% CI = 1.060–3.299, *P* < 0.001). Patients with lymph node metastasis (OR = 1.874, 95% CI = 1.444–2.431, *P* < 0.001) and higher American Association of Cancer (AJCC) stage (OR = 2.321, 95% CI = 1.788–3.012, *P* < 0.001) were observed to have higher CAR after combining 5 studies using a fixed-effect model because of low heterogeneity.

**Table 3:** Main results of meta-analysis and publication bias between CAR and clinicopathological characteristics, overall survival (OS) and progression-free survival (PFS).

Clinicopathological features/OS/PFS	Number of included datasets	OR/HR (95% CI)	Z, *P* value	Heterogeneity test (*I*^2^, *P* value)	Publication bias (egger's test) (*P* value)	Pooling model
Age (old vs. young)	5	1.122 (0.703, 1.792)	0.48, 0.629	79.3%, 0.001	0.250	Random
Sex (Male vs. Female)	7	2.181 (1.313, 3.620)	3.01, 0.003	81.7%, < 0.001	0.658	Random
Smoking status (Yes vs. No)	5	1.870 (1.060, 3.299)	2.16, < 0.001	81.9%, < 0.001	0.500	Random
Lymphatic invasion (Yes vs. No)	5	1.874 (1.444, 2.431)	4.73, < 0.001	47.2%, 0.108	0.225	Fixed
AJCC stage (III∼IV vs. I∼II)	5	2.321 (1.788, 3.012)	6.33, < 0.001	6.1%, 0.372	0.728	Fixed
OS	7	1.837 (1.532, 2.202)	6.57, < 0.001	0%, 0.902	0.752	Fixed
RFS	4	1.757 (1.376, 2.243)	4.52, < 0.001	0%, 0.465	0.394	Fixed

### Association between CAR and prognosis in patients with lung cancer

**Figure 3:**
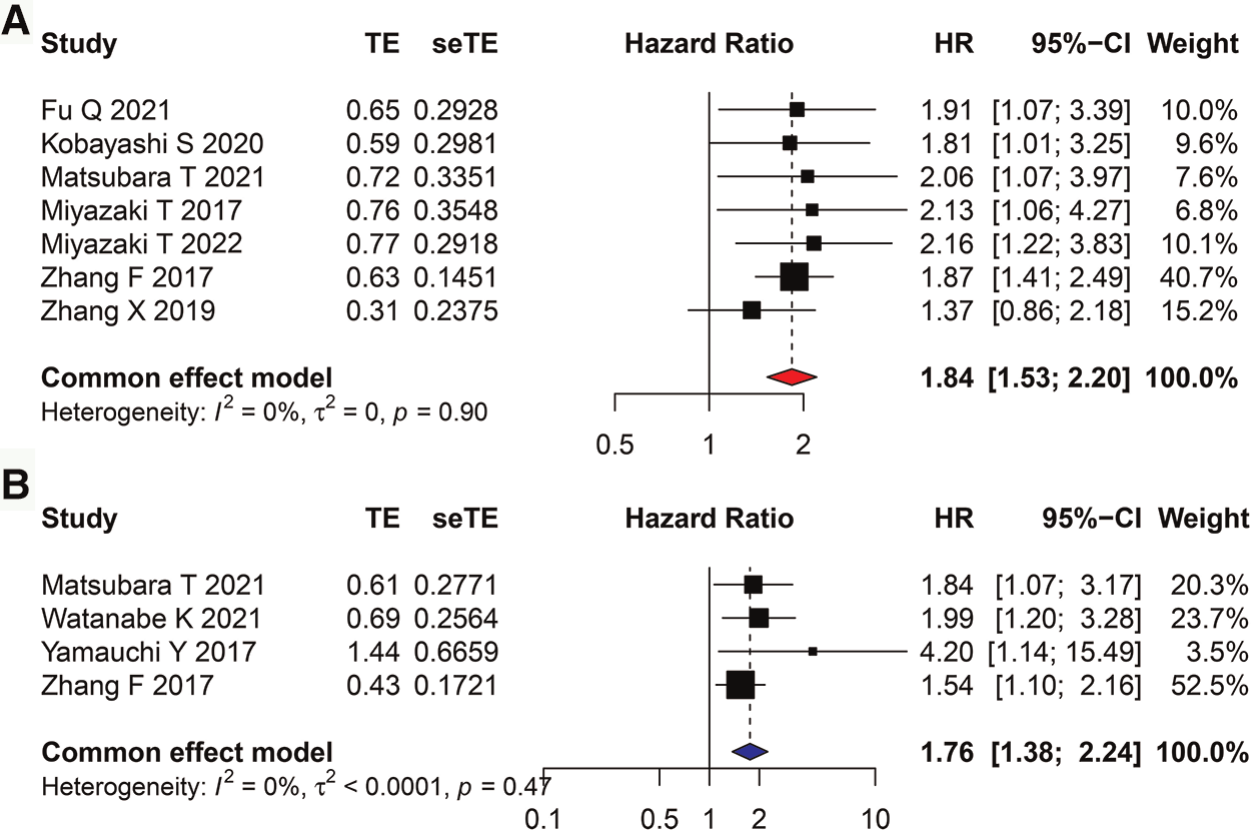
Forest plots evaluating the HRs of CAR in patients with lung cancer for (**A**) OS and (**B**) RFS. HR, hazard ratio; CAR, C-reactive protein to albumin ratio; OS, overall survival; RFS, recurrence-free survival.

The correlation between CAR and OS was assessed using 7 included studies, and that between CAR and RFS was assessed using 4 included studies. A fixed-effects model was used because of low heterogeneity. It revealed that high CAR was associated with poor OS (HR = 1.837, 95% CI = 1.532–2.202, *P* < 0.001) and poor RFS (HR = 1.757, 95% CI = 1.376–2.243, *P* < 0.001; Figures 3A,B).

### Sensitivity analyses and publication bias

**Figure 4:**
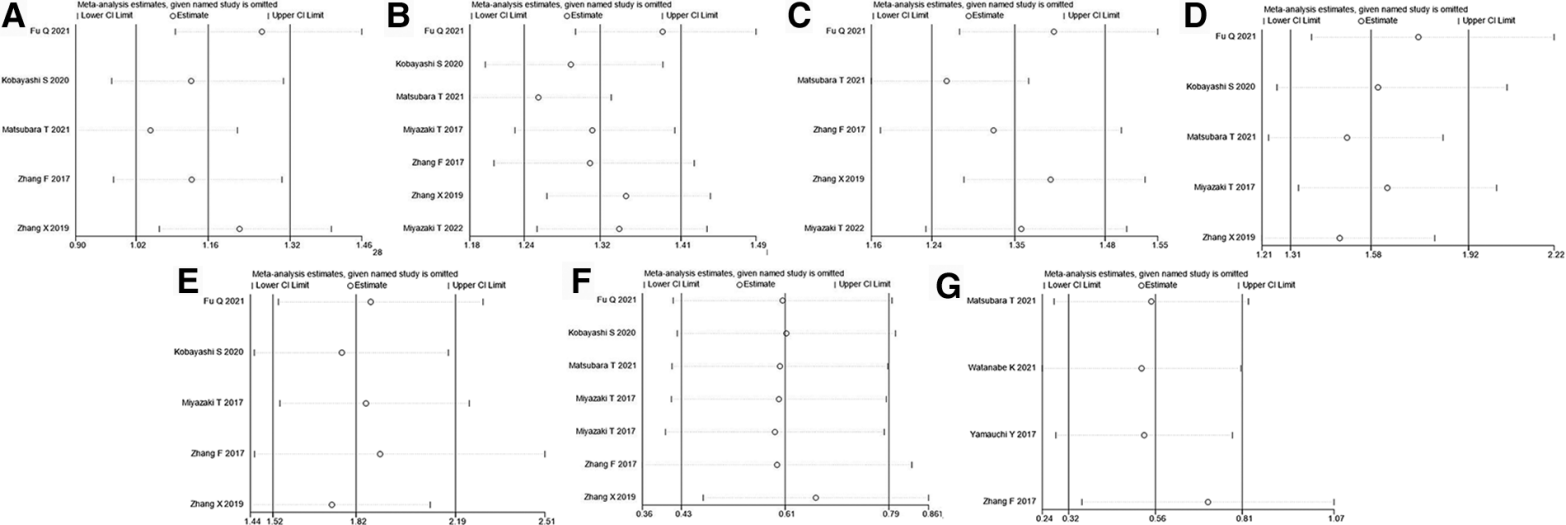
Sensitivity analysis for (**A**) age, (**B**) sex, (**C**) smoking history, (**D**) lymph node metastasis, (**E**) AJCC stage, (**F**) OS, and (**G**) RFS. AJCC, American Association of Cancer; OS, overall survival; RFS, recurrence-free survival.

**Figure 5:**
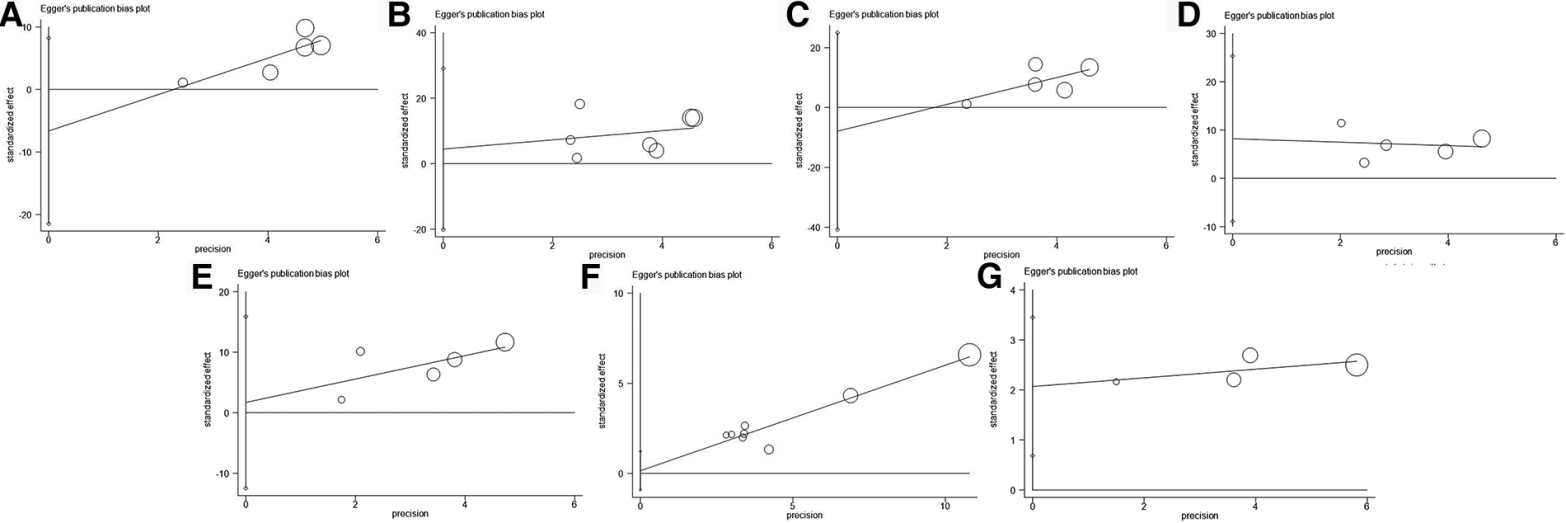
Egger's publication bias plot for (**A**) age, (**B**) sex, (**C**) smoking history, (**D**) lymph node metastasis, (**E**) AJCC stage, (**F**) OS, and (**G**) RFS. AJCC, American Association of Cancer; OS, overall survival; RFS, recurrence-free survival.

We assessed the sensitivity of this meta-analysis by deleting each study (Figures 4A–G). None of the studies significantly affected the magnitude of the combined effect after deletion, indicating that this meta-analysis provided reliable results. Furthermore, Egger's plot indicated that the distribution of most studies included in this meta-analysis was roughly symmetrical (Figures 5A–G). Combined with the corresponding calculation results in Table 3, it was shown that no publication bias existed in this meta-analysis.

## Discussion

The development of cancer and treatment outcomes of patients with cancer are usually influenced by various factors. Some researchers have hypothesized that certain stimuli can promote cell proliferation through tissue damage and inflammatory responses, and they believed that tumors develop at inflammatory sites (27). At present, some evidence indicates that tumor-related inflammatory reactions can produce oxygen free radicals and various inflammatory cytokines, which are present throughout the process of tumor occurrence and can cause changes in the tumor microenvironment (28). In addition, the survival outcome of patients with cancer largely depends on certain individual-related factors, particularly their nutritional status (29). With the continuous in-depth study of the clinical observations and patient outcomes, it is becoming more and more important to select the prognostic indicators accurately for patients with cancer. The hematological examination is one of the most common laboratory examinations in clinics because of its low cost, convenience, and rapidity. Therefore, it is expected that hematological immune-inflammatory markers would improve the predictive ability of existing prognostic tools in patients with cancer. Our study suggests that hypoalbuminemia should be treated before surgery, and some risk factors that may lead to elevated C-reactive protein should be actively treated, such as infection, thrombosis and other diseases.

CRP is an acute-phase protein produced by the liver and regulated by an inflammatory factor, namely, interleukin (IL)-6. It is a widely used, nonspecific systemic inflammatory marker (30, 31). The increase in serum CRP level in patients with cancer is correlated with the proliferation of tumor cells and production of inflammatory cells and related inflammatory factors. Moreover, preoperative CRP level has been proved to be a prognostic indicator for various cancers (32–34). Albumin is produced by hepatocytes and regulated by inflammatory cytokines, including IL-1. Previous studies have reported that albumin is mainly evaluated to assess the nutritional status of patients, can reflect the inflammatory state of the body, and is an indicator of antitumor immune response (35, 36). Malnutrition is common in patients with cancer, particularly in patients with locally advanced or metastatic malignancies, and albumin levels are generally significantly lower than those prior to the occurrence of cancer before due to excessive metabolism. In addition, hypoalbuminemia is associated with cell-mediated immune deficiency; this may reduce the systemic immune response and lead to tumor cell proliferation, which is associated with increased incidence rate of cancer and mortality in such patients (37).

Because the levels of plasma CRP and albumin are susceptible to many factors, such as water and sodium retention or dehydration, their application in determining the prognosis of patients may be limited when we use only one of these indicators. CAR not only effectively combines these two indicators but also avoids the interference of the aforementioned interfering factors. Moreover, CAR is observed to be higher in men and in patients who smoke, with lymph node metastasis, or with high AJCC stage. Moreover, these factors are some of the influencing factors of poor prognosis in patients with lung cancer. CAR, as a combination of the two indexes plasma CRP and albumin, may be used as a simple biomarker to predict the survival status of patients with lung cancer. The level of CAR in patients with lung cancer before treatment can be used as a basis for assessing the prognosis of patients and can assist in clinical decision-making. Interestingly, a meta-analysis suggested that perioperative use of nonsteroidal anti-inflammatory drugs can improve the prognosis of patients with cancer (38). This suggests that appropriate anti-inflammatory treatment would improve the prognosis of patients with lung cancer to a certain extent.

Nutritional status is currently considered to be significantly related to the prognosis of patients with lung cancer (39). Previous studies have reported that the Glasgow prognostic score (GPS) is an effective index to assess the treatment response and prognosis of end-stage lung cancer (40–42). The GPS combines biochemical analysis of albumin and CRP levels in plasma. The albumin levels and inflammation are closely related to tumor prognosis. CRP itself is a nonspecific inflammatory index. High CRP level is observed in case of not only lung cancer and end-stage tumors but also infection, inflammation, trauma, tissue injury, and stress. The increase in CRP levels in patients with end-stage cancer is mainly related to tumor progression and inflammatory response. Some studies have reported that inflammatory response can directly accelerate the catabolism of proteins in the skeletal muscle, resulting in accelerated protein degradation and nutrient loss (43). At the same time, albumin level is affected by various confounding factors; the decrease in albumin level in patients with end-stage tumors can be due to anorexia, malnutrition, gastrointestinal dysfunction, absorption disorder, tumor cachexia, dysfunction of the liver and kidney, metabolic disorder, and systemic inflammatory reaction. Therefore, these observations suggest that CRP and albumin are interrelated and influence each other.

This study has some limitations. First, all the included studies were retrospective. Although the outcome indicators of prognosis were corrected using multivariate Cox regression analysis, some confounding factors still existed that inevitably affected our results. Second, most studies included patients from Asian countries such as China and Japan, only 1 study was from Germany. Therefore, the prognostic value of CAR in NSCLC in other countries and regions remains to be studied. Third, the cutoff values of CAR in these studies were different; hence, the optimal critical value of CAR in various populations needs to be determined by performing more well-designed studies with large sample sizes. Forth, we included reports written in English or Chinese, and reports written in other languages were not included. Besides, we did not obtain gray literature, which may have caused inevitable bias.

## Conclusions

This meta-analysis emphasized that CAR is correlated with sex, smoking status, lymph node metastasis, and AJCC stage of patients with lung cancer. Moreover, higher CAR indicates a worse prognosis. In future, multicenter and high-quality studies containing a large sample size are needed to further verify our results.

## Data Availability

The original contributions presented in the study are included in the article/**Supplementary Material**, further inquiries can be directed to the corresponding author/s.
